# Associations between *P2RY12* gene polymorphisms and risks of clopidogrel resistance and adverse cardiovascular events after PCI in patients with acute coronary syndrome

**DOI:** 10.1097/MD.0000000000006553

**Published:** 2017-04-07

**Authors:** Miaonan Li, Hongju Wang, Ling Xuan, Xiaojun Shi, Tong Zhou, Ningru Zhang, Yuli Huang

**Affiliations:** Department of Cardiovascular Disease, The First Affiliated Hospital of Bengbu Medical College, Bengbu, Anhui, PR China.

**Keywords:** clopidogrel resistance, gene polymorphism, P2RY12, PCI

## Abstract

Clopidogrel resistance in patients with acute coronary syndrome (ACS) is one of the key causes of recurrent cardiovascular disease (CVD) events after percutaneous coronary intervention (PCI). Clopidogrel targets the platelet membrane receptor P2RY12 to inhibit platelet aggregation via adenosine diphosphate (ADP). This study aimed to investigate the relationships between P2RY12 polymorphisms and the risk of clopidogrel resistance and adverse CVD events after PCI. From January 2015 to December 2014, patients who had been diagnosed with ACS undergoing PCI and treated with clopidogrel were recruited for this prospective cohort study (N = 498). Data regarding demographics, medication intake, and ACS lesion were recorded, and whole blood samples were collected for biochemical tests, ADP-induced platelet aggregation ratio detection, and *P2RY12* genotyping. *P2RY12* genotyping was performed by polymerase chain reaction. The left ventricular ejection fraction was calculated by echocardiography. After 3 to 12 months of follow-up, data regarding any adverse CVD event or death were recorded. The allele frequencies for the T variation alleles in C34T and G52T of *P2RY12* were 20.3% and 11.6%, respectively. Patients with T variations at C34T or G52T of *P2RY12* had a significantly higher risk of clopidogrel resistance (C34T: *P* < 0.001; G52T: *P* = 0.003) and total cardiovascular events (C34T: *P* = 0.013; G52T: *P* = 0.018) compared to those with the wild-type genotype. Moreover, multivariable logistic regression showed that patients with the T variations in C34T (odds ratio [OR]: 2.89 (95% confidence interval [CI]: 1.48–5.64), *P* = 0.002) and G52T (OR: 3.68 [95% CI: 1.71–7.92], *P* = 0.001) also had a significantly higher risk of clopidogrel resistance. Also, the T variations in C34T (OR: 2.68 [95% CI: 1.07–6.73], *P* = 0.035) and G52T (OR: 5.64 [95% CI: 1.52–20.88], *P* = 0.010) significantly increased the risk of post-PCI CVD events after accounting for confounding factors. The *P2RY12* gene polymorphisms C34T and G52T were significantly associated with a higher risk of clopidogrel resistance and sequential cardiovascular events in Chinese ACS patients after PCI.

## 1 Introduction

Cardiovascular disease (CVD) has now become the leading cause of death in China,^[1]^ and the related burden is expected to continuously increase over the next few decades.^[2]^ Acute coronary syndrome (ACS), a severe type of CVD, leads to arrhythmia, heart failure, and even sudden death. The application of percutaneous coronary intervention (PCI) combined with antiaggregation treatment with aspirin and clopidogrel has markedly improved the prognosis of ACS.^[3,4]^ However, some patients are susceptible to recurrent CVD events after comprehensive intervention, including cardiac death, in-stent restenosis, and stent thrombosis.^[5,6]^ Dual antiplatelet therapy with clopidogrel and aspirin is currently recommended for patients with coronary artery disease (CAD) and in those undergoing coronary stent implantation. In approximately 5% to 30% of clopidogrel-treated patients, the inhibition of platelet aggregation is insufficient.^[7]^ As a result, patients with clopidogrel resistance may have a higher risk of recurrent CVD events. Genetic polymorphisms that influence the response to clopidogrel may encode the proteins that are responsible for clopidogrel absorption, its biotransformation to the active form, and the platelet drug receptor.^[8]^ The *P2RY12* gene encodes the adenosine diphosphate (ADP) receptor P2Y12, the pharmacological target of clopidogrel. Common variation in the *P2RY12* gene has been suggested as one of the mechanisms underlying this large variability in clopidogrel response.^[7]^ Recently, a clinical study reported that 18C > T SNP of the *P2RY12* gene may be an independent predictor of pharmacological response to clopidogrel,^[9]^ whereas 2 other studies failed to demonstrate any association between a sequence variation (T744C) of *P2RY12* gene and platelet response to clopidogrel in patients with ACS.^[10,11]^ However, there are still limited data regarding the effects of *P2RY12* gene polymorphisms on clopidogrel resistance. Moreover, studies about the relationships between *P2RY12* gene polymorphism and cardiovascular health outcome are scarce. Therefore, this study aimed to investigate the relationships between C34T, G52T in *P2RY12* and the risk of clopidogrel resistance, and sequential CVD events after PCI in Chinese ACS patients.

## 2 Methods

### 2.1 Patients

Patients who were diagnosed with ACS and subsequently underwent PCI between January 2015 and December 2015 in the Cardiovascular Department, The First Affiliated Hospital of Bengbu Medical College, Bengbu, Anhui Province, China were recruited. All participants were Han Chinese. The exclusion criteria included a previous history of ACS or PCI, medical use of clopidogrel before admission, moderate to severe valve disease, liver or kidney failure, history of hematopoietic diseases, infectious disease, tumor, or other types of consumptive diseases. A total of 498 patients (310 males and 188 females) were included in the final analysis. All participants signed consent forms before the study. This study was approved by the ethics community of the First Affiliated Hospital of Bengbu Medical College, Bengbu, Anhui Province, China.

### 2.2 Study procedure

#### 2.2.1 Presurgery evaluation and medication

All patients were under intensive care and underwent a comprehensive evaluation. Demographic data including age, gender, and smoking status were collected upon admission. Current smoker was defined by a habit of smoking on a regular basis. All patients were given aspirin and 300 mg clopidogrel (loading dose) from the day of admission. If no contraindication was found, 100 mg aspirin and 75 mg clopidogrel were given per day as the maintenance dose. Five-milliliter samples of whole blood were collected both before and 7 days after the intake of medications for examination of the ADP-induced platelet aggregation ratio (PAR). Whole blood was also collected on the second day of admission for biochemical tests and genotyping.

#### 2.2.2 Percutaneous coronary intervention

PCI was performed by cardiovascular specialists. Coronary arteriography was performed, and the results were judged based on the 2001 ACC/AHA criteria.^[12]^ A drug-eluting stent was placed if the arterial blockage by a lesion was more than 70%. Success of the stent insertion was defined by less than 20% residual blockage and thrombolysis in myocardial infarction 3 flow. Gensini^[13]^ score was calculated for the quantification of the coronary arterial blockage improvement of each lesion by 2 professionally trained cardiovascular specialists separately. The average Gensini score was used in the final analysis.

#### 2.2.3 Perisurgery medication and follow-up survey

Routine use of aspirin and clopidogrel was continued throughout PCI surgery for all patients. Other medications included low-molecular-weight heparin, isosorbide mononitrate, and rosuvastatin. Benazepril and metoprolol tartrate sustained release tablets were used for the adjustment of blood pressure and heart rate in some patients. Pantoprazole was used if patients had gastrointestinal symptoms. All patients were followed for 3 to 12 months (follow-up ended in March 2016) after discharge by telephone or outpatient service. Information such as adverse cardiovascular events was collected at follow-up. During follow-up, a total of 3 patients died. One patient died of respiratory failure caused by lung infection, 1 died of myocardial infarction, and the other one died of heart failure.

### 2.3 Biochemical tests

Fasting plasma glucose, total cholesterol, triglycerides, low-density lipoprotein cholesterol, high-density lipoprotein cholesterol, serum creatinine, liver enzymes including aspartate aminotransferase (AST) and alanine aminotransferase, uric acid, and C reactive protein levels were tested using an Olympus AU5800 autoanalyzer (Olympus Co., Tokyo, Japan). The PAR was examined using an AggRAM system (Helena Laboratories, Beaumont, TX).

### 2.4 Echocardiography

Transthoracic bedside echocardiography was performed by 2 professionally trained echocardiography specialists separately. Patients lay on their left side when echocardiography was performed on the third and fourth intercostal space. The heart was then visualized in the left parasternal long axis, and the left ventricle was visualized by M mode echocardiography. The left ventricular ejection fraction (LVEF) was then measured.

### 2.5 DNA extraction and genotyping

Deoxyribonucleic acid (DNA) was extracted from whole blood samples using a DNA extraction kit according to the manufacturer's instructions (TIANGEN, Beijing, China). Polymerase chain reaction (PCR) analysis was then performed using PCR purification reagents (PrimeSTAR HS [Premix], TaKaRa, Dalian, China). The forward primer sequence for *P2RY12* was GGTAACCAACAAGAAATGCAAGC, and the reverse sequence was GGACAGTGTAGAGCAGTGGGAAG. Each 20-μL PCR mixture contained 5 μL 5× PrimeSTAR buffer, 2 μL dNTP mixture, 1 μL primer, 0.2 μL Taq polymerase, 2 μL DNA template, and 9.8 μL distilled water. The PCR program consisted of 5 minutes at 95 °C for denaturation, 30 cycles of 10 seconds at 95 °C, 15 seconds at 60 °C, 25 seconds at 75 °C, and followed by 10 minutes at 70 °C for extension and 30 seconds at 40 °C for annealing. Genotyping was performed by GENEWIZ Co. (Suzhou, China).

### 2.6 Definitions and major outcomes

In this study, ACS was diagnosed if the patient had either unstable angina pectoris, non-segment elevated myocardial infarction (non-STEMI), or STEMI.^[14]^ The major outcome of this study was clopidogrel resistance, which was defined as a less than 10% change in the PAR after the intake of clopidogrel for 7 days.^[15]^ The secondary outcome was adverse cardiovascular events, which included recurrent angina, acute myocardial infarction, emergency revascularization, in-stent restenosis, stent thrombosis, and mortality.

### 2.7 Statistical analysis

All statistical analyses were performed using SPSS 17.0 software (SPSS Inc., Chicago, IL). No patient had a missing value, and all patients finished complete follow-up. Quantitative data are presented as mean ± standard deviations, and categorical data as the number and proportion. Student *t* test was performed for comparison between different genotypes. Fisher exact test was performed for comparisons of proportions. Pearson test was performed for covariate preselection. Relationships between genotypes and clinical outcome were evaluated by multivariable logistic regression using the backward method. A 2-tailed *P* value less than 0.05 was considered to be statistically significant.

## 3 Results

### 3.1 P2RY12 genotype frequencies

Among the 498 patients, genotyping results showed that 324 patients had CC (65.1%), 146 had CT (29.3%), and 28 had TT (5.6%) at C34T of *P2RY12*. The allele frequencies for the C and T alleles were 79.7% and 20.3%, respectively. At G52T, 396 patients had GG (79.5%), 88 had GT (17.7%), and 14 had TT (2.8%). The allele frequencies for the G and T alleles were 88.4% and 11.6%, respectively. Both polymorphisms were in Hardy–Weinberg equilibrium. Patients with a gene variation of the T variation either in the heterozygote or homozygote group were compared to patients with the wild-type genotype.

### 3.2 Baseline characteristics

The baseline characteristics of patients are presented in Table 1. No differences were found in age, gender proportion, smoking habit, biochemical results, LVEF, medication intake, CADs, or Gensini score between the different genotype groups. However, patients with a variation at C34T or G52T of *P2RY12* had a significantly higher risk of clopidogrel resistance (C34T: *P* < 0.001; G52T: *P* = 0.003) and total cardiovascular events (C34T: *P* = 0.013; G52T: *P* = 0.018) compared to the wild-type genotype (Table 1).

**Table 1:**
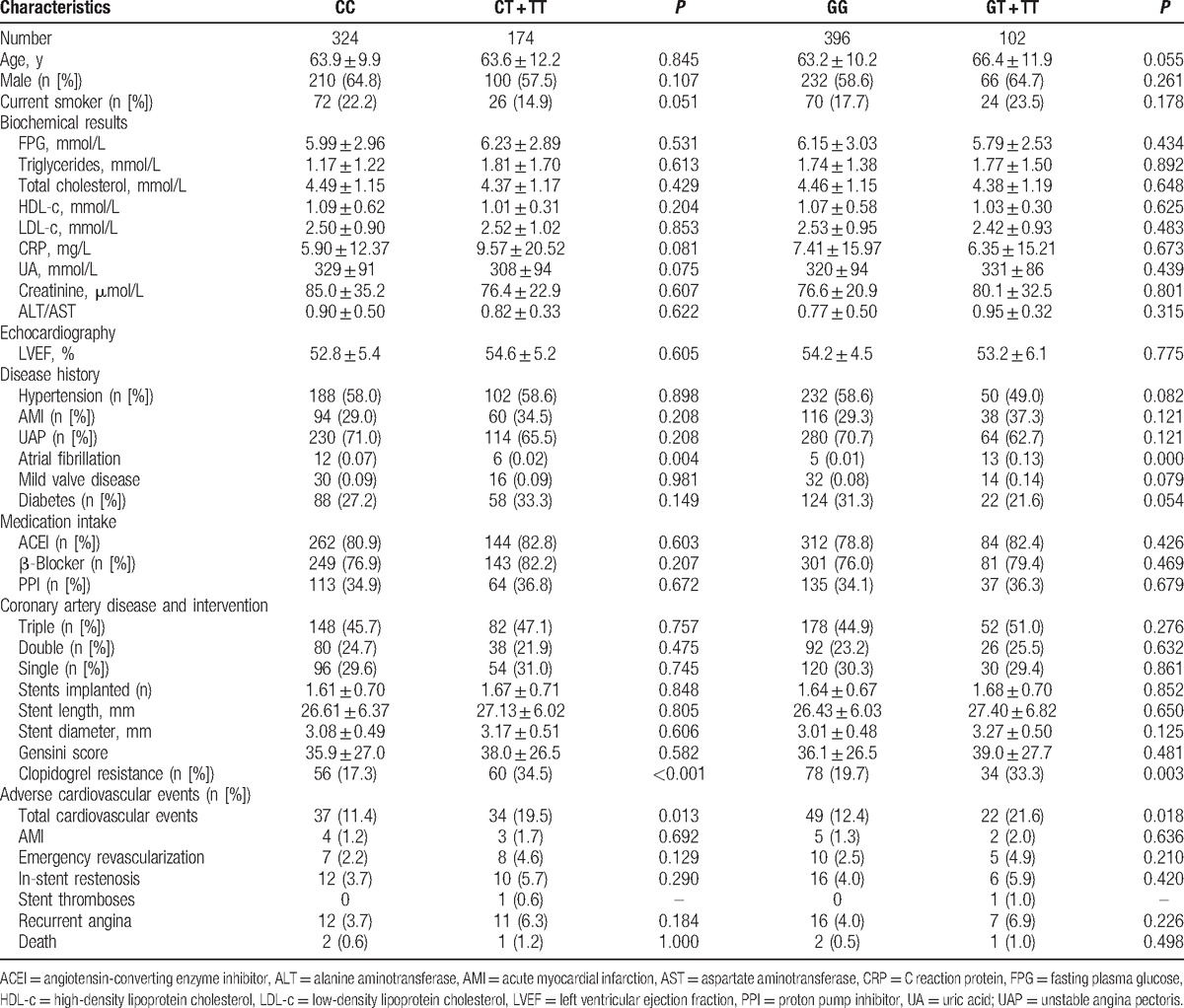
Baseline characteristics in different *P2RY12* genotype groups.

### 3.3 Relationships between P2RY12 polymorphisms and risk of clopidogrel resistance

The relationships between the *P2RY12* polymorphisms and the risk of clopidogrel resistance are shown in Table 2. Multivariable logistic regression showed that patients with the T variation at C34T had a significantly higher risk of clopidogrel resistance than patients with the CC genotype (odds ratio [OR]: 2.89 (95% confidence interval [CI]: 1.48–5.64), *P* = 0.002), and the T variation at G52T also significantly increased the risk by 3.7 times (OR: 3.68 [95% CI: 1.71–7.92], *P* = 0.001). These associations did not depend on patient age, gender, smoking status, hypertension, diabetes, or any biochemical results (Table 2).

**Table 2:**
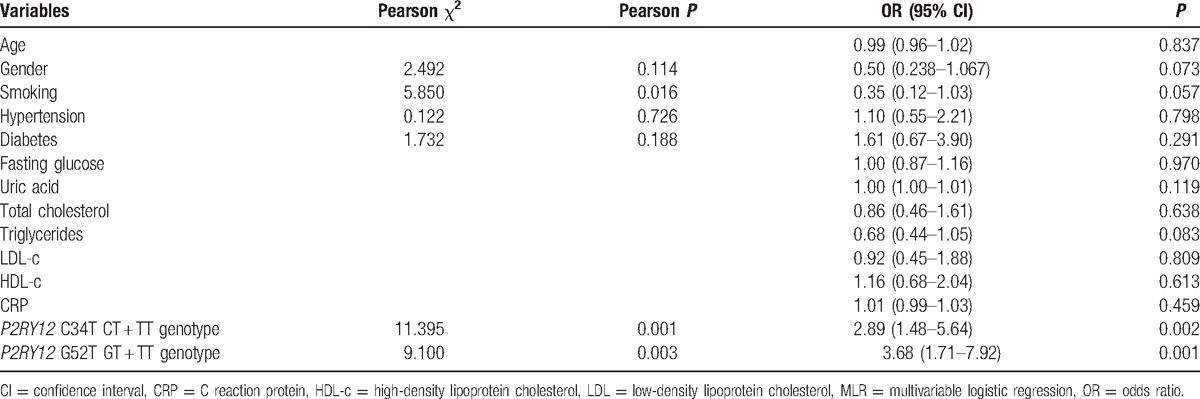
Relationships between patient characteristics and risk of clopidogrel resistance.

### 3.4 Relationships between P2RY12 polymorphisms and risk of adverse cardiovascular events

The relationships between *P2RY12* polymorphisms and the risk of adverse CVD events are presented in Table 3. After adjusting for age, gender, smoking status, hypertension, diabetes, biochemical results, and clopidogrel resistance, the T variation in C34T significantly increased the risk of post-PCI CVD events (OR: 2.68 [95% CI: 1.07–6.73], *P* = 0.035). However, the T variation at G52T significantly increased the CVD risk after accounting for confounding factors (OR: 5.64 [95% CI: 1.52–20.88], *P* = 0.010).

**Table 3:**
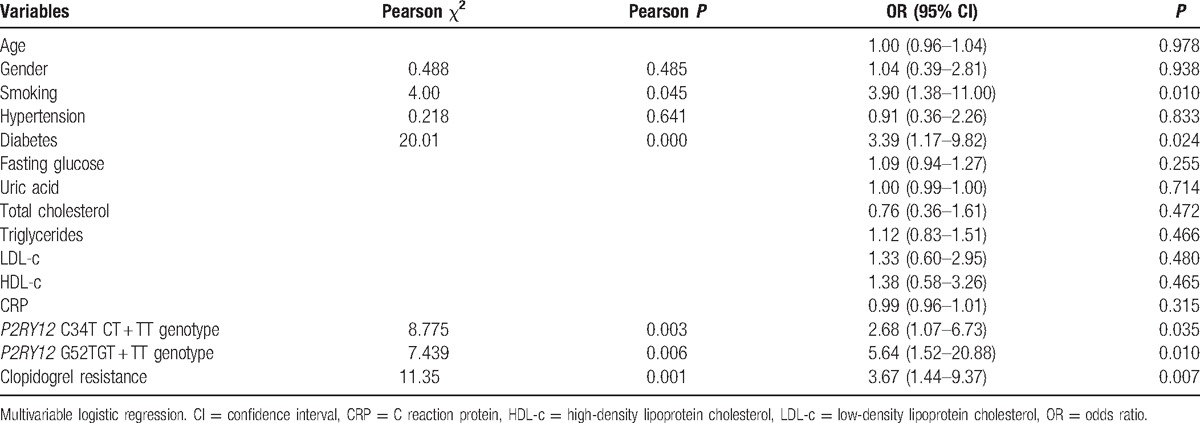
Relationships between patient characteristics and risk of total adverse cardiovascular events.

## 4 Discussion

This cohort study demonstrated that variations at C34T and G52T of the *P2RY12* gene significantly increased the risk for clopidogrel resistance in Chinese ACS patients. The findings also suggested that patients with C34T or G52T polymorphism had a higher risk of adverse cardiovascular events after PCI.

The 2 most widely used antiplatelet drugs in the world are aspirin and clopidogrel. Both are efficient for secondary prevention of CVDs through different mechanisms of action. Nevertheless, the CURE trial found that dual antiplatelet therapy with clopidogrel and aspirin in ACS reduced adverse coronary events by 20% when compared with aspirin monotherapy.^[16]^ The results of the present study showed a 3.67 times higher risk of post-PCI CVD events in patients with clopidogrel resistance. It has been suggested that the risk of antiplatelet therapy failure in dual antiplatelet therapy may be greatly increased if clopidogrel resistance occurs. Moreover, the CAPRIE study revealed the modest superiority of clopidogrel monotherapy over aspirin monotherapy.^[17]^ These findings not only demonstrate that clopidogrel has superior effectiveness to aspirin in antiplatelet monotherapy, but that it also plays a key role in dual antiplatelet therapy. If patients have a poor or no response to clopidogrel treatment, the inhibition of platelet aggregation by aspirin only may be insufficient. As a result, patients may have a higher risk of recurrent CVD events.

Previous studies have reported associations between gene polymorphisms and clopidogrel resistance. The cytochrome P450 (CYP) enzyme gene polymorphism has been the most studied gene in this respect, and its variation at *CYP2C19* is significantly associated with a lower response to clopidogrel.^[18–20]^ However, previous studies on the *CYP2C19* gene found it could only explain 12% of the variability in clopidogrel resistance, indicating that other gene polymorphisms might also play important roles. Our findings support the notion that patients with T variations at C34T or G52T of *P2RY12* had a significantly higher risk of clopidogrel resistance (C34T: *P* < 0.001; G52T: *P* = 0.003) compared to those with the wild-type genotype (Table 1). Moreover, the variability of *P2RY12* in clopidogrel resistance was much larger both at C34T (34.94%) or G52T (20.48%), which indicated that *P2RY12* polymorphisms may play an important role in the mechanisms of clopidogrel resistance. Previous studies on the T744C polymorphism in the *P2RY12* gene found no significant association with clopidogrel response.^[10,21,22]^ Nevertheless, our study investigated the significant associations between platelet receptor *P2RY12* gene polymorphisms and clopidogrel resistance, which adds very valuable information in this field. These results were consistent with those of a small study in Turkey that also demonstrated an increased risk of clopidogrel resistance with the C34T variation of *P2RY12*.^[23]^ However, a cohort study conducted by Tang et al^[8]^ in Han Chinese suggested that the effect of C34T genotype alone on clopidogrel response was small. In our study, we did not include the *CYP2C19* genotyping; therefore, it remains possible that *CYP2C19* polymorphism may also play a role. Clopidogrel exerts its antiaggregating effect by irreversibly binding to P2RY12. Therefore, the potential mechanism of these gene variations and clopidogrel resistance found in our study might be due to their affected binding affinity and/or postreceptor signaling pathways. However, further studies on the mechanism of platelet receptor-related clopidogrel resistance are warranted.

Inadequate blockage of platelet aggregation in ACS patients can lead to recurrent CVD events after PCI. Previous studies have also suggested association between gene polymorphisms and recurrent CVD. The *CYP2C19 681* G > A mutation, which is associated with reduced clopidogrel antiplatelet activity, is an important marker for poor prognosis in ACS patients receiving clopidogrel treatment.^[24]^ Moreover, a recent meta-analysis, carried out on nearly 8000 patients with CAD undergoing clopidogrel treatment, showed that the *CYP2C19∗2* polymorphism is associated with an increased risk of major adverse cardiovascular events and stent thrombosis.^[25]^ However, little has been reported about the relationship between *P2RY12* gene polymorphisms and the risks of adverse CVD events after PCI. We have shown for the first time that patients with T variations at C34T or G52T of *P2RY12* had a significantly higher risk of total cardiovascular events (C34T: *P* = 0.013; G52T: *P* = 0.018) compared to those with the wild-type genotype. More recently, 2 studies demonstrated that a genetic defect of *CYP2C19* led to an approximately 2 times higher risk of subsequent cardiovascular events, including nonfatal myocardial infarction and stroke.^[26,27]^ Our study also demonstrated that T variations at the C34T and G52T sites of *P2RY12* were associated with a 2.7 and 5.6 times greater risk for CVD events after PCI, respectively. Hence, our data have demonstrated that gene polymorphisms are associated with clopidogrel resistance and, in turn, significantly increased the risk of post-PCI CVD events. Other genetic and environmental factors may also play critical roles, which require further analysis.

In addition to the genetic effect on clopidogrel resistance and the prognosis of ACS, other factors including obesity,^[28]^ diabetes,^[29]^ smoking habit,^[30]^ and drug interaction^[31]^ also have been reported to be influential factors. Based on our data, we also found that smoking and diabetes were significantly associated with post-PCI CVD events, suggesting the important role of lifestyle and diabetes on post-PCI CVD health besides the demonstrated genetic effect.

Our study also had several limitations. First, the perspective study only had 1 time point for follow-up; therefore, a survival test could not be performed. Second, only dominant model was used in our analysis due to sample size limitation. Third, a single assessment of platelet function and only 1 method for testing platelet function may not be sufficient to fully diagnose the response to antiplatelet therapy. Fourth, there may be some clinical factors that are difficult to control, such as low drug adherence. Clopidogrel therapy may fail due to patient noncompliance with prescriptions from physicians. In addition, there may be variability in absorption with associated underdosing in patients, possible drug–drug interactions, and possible effects of other drug effects, such as herbal medicine taken at the same time, on prognosis.

## 5 Conclusion

Our study demonstrated that *P2RY12* polymorphisms were associated with higher risks of clopidogrel resistance and adverse cardiovascular events after PCI in Chinese ACS patients. More clinical and laboratory studies are required for further elucidation of the mechanisms of clopidogrel response variation, especially the function of P2RY12 in antiaggregation treatment. In order to optimize the effect of antiaggregation treatment and reduce the risk of recurrent cardiovascular events, a personalized regimen for those carrying T gene at C34T and G52T of *P2RY12* may be warranted.
